# Comparative analysis of microbial contamination in diesel fuels using MALDI-TOF MS

**DOI:** 10.1038/s41598-025-87713-1

**Published:** 2025-02-06

**Authors:** Agnieszka Ludwiczak, Tomasz Zieliński, Ewelina Sibińska, Grażyna Czeszewska-Rosiak, Michał Złoch, Joanna Rudnicka, Andrzej Tretyn, Paweł Pomastowski

**Affiliations:** 1 Centre for Modern Interdisciplinary Technologies, Nicolaus Copernicus University in Torun, Wilenska 4 Str, Torun, 87-100 Poland; 2 Department of Immunology, Faculty of Biological and Veterinary Sciences, Nicolaus Copernicus University in Torun, Lwowska 1 Str, Torun, 87-100 Poland; 3 Chair of Plant Physiology and Biotechnology, Nicolaus Copernicus University in Torun, Lwowska 1 Str, Torun, 87-100 Poland

**Keywords:** MALDI-TOF MS, Diesel fuel, Microbial contamination, Zybio, Bruker, Actinomycetia, Microbiology, Chemistry

## Abstract

Microbiological contamination in fuels poses a significant threat to fuel quality, operational safety, and the efficiency of fuel systems. Effective management of microbial risks can extend fuel systems’ lifespan and minimize the environmental footprint of fuel usage. Therefore, we aimed to identify the microbiological contaminants using two MALDI-TOF MS systems (Bruker Daltonik GmbH and Zybio Inc.). Detection and characterization of microbiological contamination in fuels were performed with further validation by 16 S rRNA gene sequencing. Microorganisms collected from three types of diesel fuel were cultured, and fuel samples were analyzed. Significant differences in microbiological contamination were observed between the fuel types, with 272 isolates of bacteria and fungi identified across all fuel samples. Ekodiesel Ultra B0 had the highest number of identified microorganisms. The Zybio system identified 48% of all microorganisms at the species level, whereas the Bruker system identified only 33%. The 16 S rDNA sequencing confirmed proteomic identifications by the MALDI systems with high accuracy. Our study indicates that MALDI-TOF MS technology can effectively assess microbiological contamination in fuels. Integrating MALDI-TOF MS with other molecular techniques could provide a comprehensive view of fuel’s microbial communities.

**Supplementary Information:**

The online version contains supplementary material available at 10.1038/s41598-025-87713-1.

## Introduction

Modern diesel engine technologies place particularly high demands on fuel quality. The innovative technological solutions in the car engine have made even the smallest fuel contaminants capable of causing significant operational problems and reducing engine longevity^1,2^. Fuel contaminants have various origins. Production processes such as oil refining, as well as the transportation and storage of fuels, can be sources of particulate matter originating from the corrosion of tanks, pipes, and other elements of the transportation and storage infrastructure. Water is another contaminant that enters the fuel through the condensation of water vapor in fuel tanks, especially under fluctuating temperature conditions. This induces the growth of microorganisms^3,4^.

Microbiological contamination in fuels primarily involves the growth of bacteria, fungi, and yeasts that can cause various issues including fuel degradation, filter blockages, and corrosion^5,6^. In addition, the microorganism’s growth in the presence of water and organic matter leads to the formation of biofilms that can significantly impact fuel quality and system integrity. The proliferation of microorganisms in fuels is particularly pronounced during extended storage periods^1,7^. Fuel contaminants can lead to incomplete combustion in the combustion chamber resulting in higher emissions of pollutants like nitrogen oxides (NOx), carbon monoxide (CO), and particulate matter (PM)^8^. These pollutants contribute to air pollution, negatively impacting air quality and public health. Contaminants can also lead to the formation of carbon deposits on engine components, intensified the problems of incomplete combustion and contributing to long-term engine issues^9^. The impact of microorganisms on the reduction of diesel fuel quality through induced degradation is also significant. The growth and proliferation of microorganisms lead to the production of various metabolic byproducts, such as organic acids, alcohols, and gases. These substances can adversely affect the fuel and components of the fuel system by contribution with the corrosion of metal parts in tanks and fuel systems and decrease the chemical stability of the fuel^2,9^.

Microbiological contamination in fuels can significantly impact fuel quality, operational safety, and the durability and efficiency of fuel systems. By acids production stimulate the corrosion of metal components in tanks and fuel systems, leading to premature failures and the need for replacement of parts or entire engine systems^6,8^. The activity of the microorganisms in closed fuel tanks results in the formation of gelatinous oil sludge, which reduce the quality of the final product. Additionally, the formation of sticky deposits and biofilms can clog fuel filters and injector nozzles, resulting in reduced engine performance and increased fuel consumption^2,9^. Therefore, the effective management of microbiological risks in fuels is essential for maintaining safety, reliability, and operational efficiency in the energy and transportation sectors, as well as in areas where fuels are a critical infrastructure element.

Various species of microorganisms, including bacteria, fungi (yeasts and moulds), and even microalgae, have been identified in fuel tanks and fuel^7,8^. Among the bacterial community, the most commonly found bacteria are *Pseudomonas* spp., *Sphingomonas* spp., *Acinetobacter* spp., *Desulfovibrio* spp., and *Clostridium* spp. *Pseudomonas* are aerobic bacteria with versatile metabolic capabilities. They exhibit the ability to produce biosurfactants, which can cause fuel emulsification, leading to issues with fuel quality and stability^10^. Similar to *Acinetobacter*, they can lead to the formation of deposits and biofilms that clog fuel filters and fuel lines. Sulfate-reducing bacteria, particularly from the genus *Desulfovibrio*, can induce anaerobic corrosion by producing hydrogen sulphide^7,11^.

Various methods are employed to monitor the levels of microbiological contamination in liquid fuels. One of the most common techniques is ATP (adenosine triphosphate) testing, which uses bioluminescence to quickly determine the overall biological activity in a fuel sample (ASTM D7463-08 standard)^12^. Despite its sensitivity, this method does not detect microbiological contaminants in the early stages. It is also not specific to microorganisms in the fuel and can detect other sources of ATP occurring in the sample. A high ATP content in the sample indicates the presence of active microorganisms, however the method does not provide information about their taxonomy^7^. Another method is the microbial impedance method. These methods allow for the rapid assessment of microbiological risk in fuels by measuring changes in the electrical conductivity of the medium caused by the growth of microorganisms^13^. However, due to their ability to identify and quantify various types of microorganisms, culture-based methods remain the standard for diagnosing microbiological contamination.

The aim of our study was to identify microbiological contaminants responsible for sediment formation affecting the dispersive stability of three types of diesel fuels in storage tanks at distribution stations. The new aspect of our research is the investigation of two different MALDI-TOF MS systems (Zybio EXS 2600 and Bruker MALDI Biotyper 4.1) in assessing microbiological contamination for fuel contamination evaluation. In addition, the proteomic technique was compared with the genetic approach (16s rRNA sequencing) to determine identification consistency. MALDI (Matrix-Assisted Laser Desorption/Ionization) as an advanced mass spectrometry technique was applied in various scientific fields^14,15^. Compared to PCR, focusing on the genetic profiles, MALDI relies on protein profiles of identified microorganisms. As a powerful tool for the rapid and accurate identification of microorganisms, MALDI is increasingly used in microbiological diagnostics, including quality control of pharmaceutical products and food^16,17^ and is still being researched for new applications.

## Results

### Microbiological contamination of diesel fuels

Microbiological contamination of diesel fuels was evidenced by the clearly visible turbidity and the presence of sediments in the samples (Supp. Fig. 1). A total of 272 isolates of bacteria and fungi were identified from the 16 diesel fuel samples, including only two genera of fungi: *Naganishia* and *Rhodotorula*. Aerobic or facultatively anaerobic bacteria predominated among the microorganisms, with capnophiles constituting only 1.8%. The chi-square test indicated statistically significant differences in the number of cultured bacteria across the different media types (p-value ≈ 4.95 × 10^− 26^) (Supp. Fig. 3a). The best bacteria growth was noted for Schedler medium (33.82% of identified bacteria), with good growth also observed on China Blue (18.01%), TSA (20.96%), and TYEA (23.16%) media. The least bacterial growth was noticed for BHA (2.94%) and M9 (1.10%) media. Among the identified microorganisms, Gram-positive bacteria predominated (92.36%), while Gram-negative bacteria accounted for just over 6% (Supp. Fig. 3b).

**Fig. 1:**
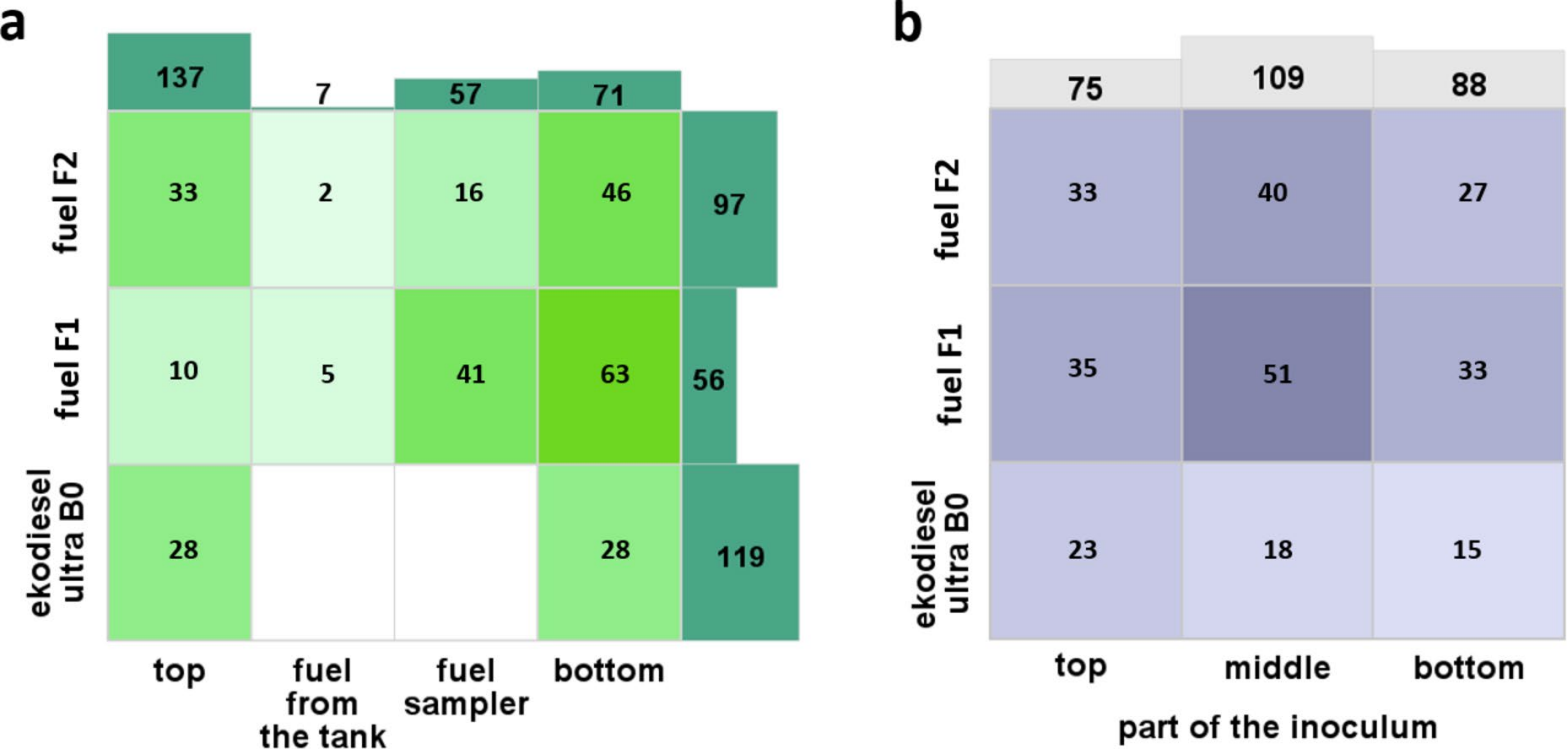
The number of identified microorganisms for different types of fuel depending on the collection level from the fuel tanks (a) and the batch of inoculum used for bacteria culturing (b).

The highest number of microorganisms was identified in tank storage Ekodiesel Ultra B0 fuel (*N* = 119), while the lowest number was found in Fuel F1 (*N* = 56) (Fig. 1a). Interestingly, the most microorganisms were identified in the upper part of the fuel tanks (*N* = 137), with the least found in fuel obtained from the tank before it was emptied for cleaning (*N* = 7). Among the three types of analyzed fuels, the most microorganisms were identified in the bottom part of the Fuel F1 tank (*N* = 63), and significantly lower were found in the bottom part of the Fuel F2 tank (*N* = 46). Notably, 35% of the microorganisms identified in the Fuel F2 tank were found in the upper part of the tank. An equal number of microorganisms were identified from both the upper and bottom parts of the tank containing the Ekodiesel Ultra B0 fuel (*N* = 28). The most prolific sources of inoculum were the middle parts of the Fuel F1 (*N* = 51) and (*N* = 40) fuel samples, as well as the upper inoculum of Ekodiesel Ultra B0 fuel (*N* = 23) (Fig. 1b).

## MALDI identification levels in fuel samples

**Fig. 2:**
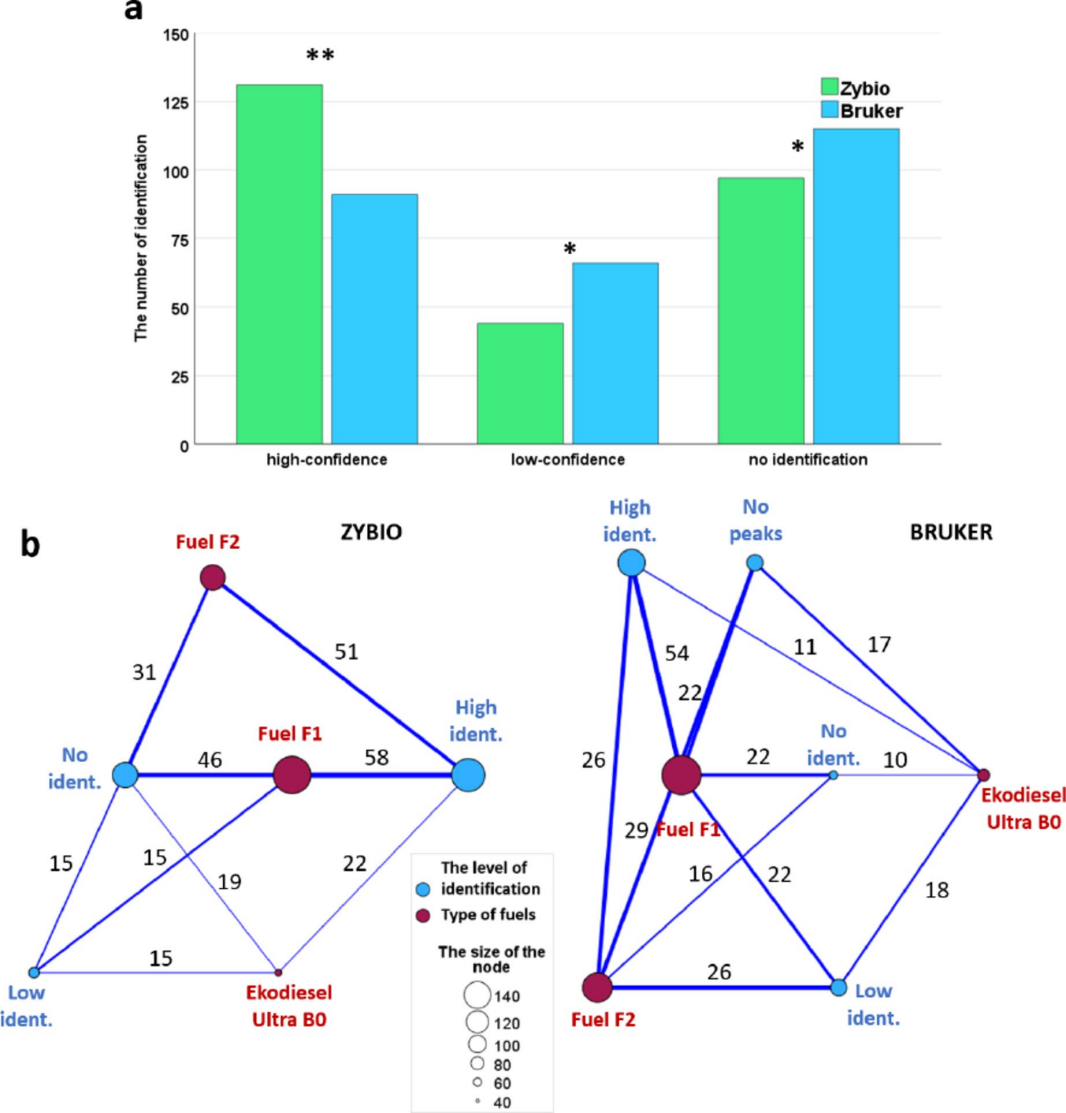
Identification levels for two MALDI systems (a) with a difference map showing the distribution of identification levels between fuel type (b). (a) The adopted identification levels are: high-confidence (score ≥ 2.000), low-confidence (1.700 ≤ score ≤ 1.999), no identification (score < 1.699), and no peaks found. Asterisks indicate levels of statistical significance: *≥0.05, **≥0.01. (b) The sizes of the nodes correspond to the number of microorganism identifications in different types of fuels (red nodes) and different identification levels (blue nodes). The thickness of the connections between nodes correlates with the number of identifications, which are noted along the connections.

The Wilcoxon test revealed a statistically significant difference in the types of identifications depending on the two MALDI systems used for analysis (Fig. 2a). The Zybio system identified 48% of all microorganisms at the species level, whereas the Bruker system identified 33%. Contrary, genus-level identification was more effective with the Bruker system. Two times more samples were unidentified by the Zybio system compare with Bruker. Additionally, the Bruker system failed to find signals in 25% of the samples submitted for identification. The frequencies of different identification levels performed by the two MALDI systems varied for each type of fuel (Fig. 2b). The highest identification rates in the Zybio system were for samples from fuel F1 (*N* = 58) and Fuel F2 (*N* = 51). However, the highest number of unidentified microorganisms were coming from the fuel F1 (*N* = 46). The lowest non-identifications were recorded for microbiological samples from Ekodiesel Ultra B0 (*N* = 19). The Bruker system most frequently assigned high-confidence scores to samples from fuel F1 (*N* = 54). Among the three analyzed fuel types, samples from Ekodiesel Ultra B0 had the lowest rates of non-identification (no identification *N* = 10, no peaks found *N* = 17).

## Bacterial classes in fuel samples identified by the Zybio and Bruker systems

**Fig. 3:**
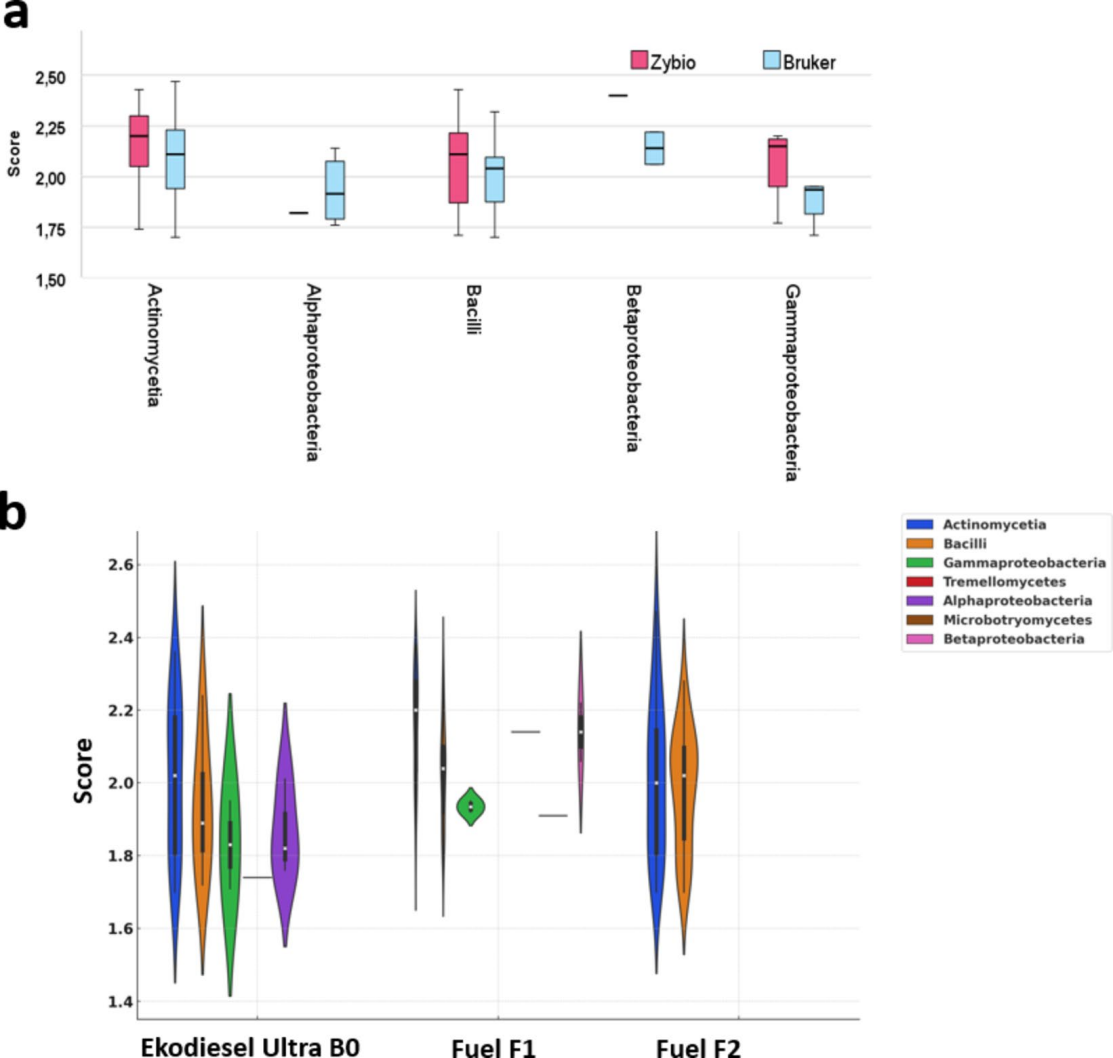
Distribution of score values for identified bacterial classes based on (a) identification by two MALDI systems (Zybio and Bruker) and (b) types of fuels.

A total of 63% and 57% of all identified microorganisms were recognized at the genus and species levels by the Zybio and Bruker systems, respectively. The Zybio system classified microorganisms into three classes: *Actinomycetia* (*N* = 70), *Bacilli* (*N* = 52), and *Gammaproteobacteria* (*N* = 5). The Bruker system identified five classes: *Actinomycetia* (*N* = 49), *Bacilli* (*N* = 38), *Alphaproteobacteria* (*N* = 2), *Gammaproteobacteria* (*N* = 2), and *Betaproteobacteria* (*N* = 2) (Fig. 3a). The Mann-Whitney test revealed statistically significant differences in the scores between the two identification systems for the *Actinomycetia* and *Bacilli* classes. The most diverse fuel type in terms of microorganism classes was the fuel F1, from which *Actinomycetia* and *Bacilli* were identified as the two most numerous classes (*N* = 35 each) (Suppl. Figure 4). Only two classes (*Actinomycetia* and *Bacilli*, *N* = 26) were present in the Fuel F2.

The fuel types also differed in the score distribution profiles for the respective classes, as shown for the Bruker system (Fig. 3a). For the *Actinomycetia* class, which is present in all fuel types, different score distributions were observed depending on the fuel type, with the most similar score distributions noted for Ekodiesel ultra B0 and Fuel F2. More varied score values in the *Gammaproteobacteria* class were observed for samples from Ekodiesel Ultra B0 compared to those from fuel (Fig. 3b).

## Bacterial species in fuel samples identified by two MALDI systems

**Fig. 4:**
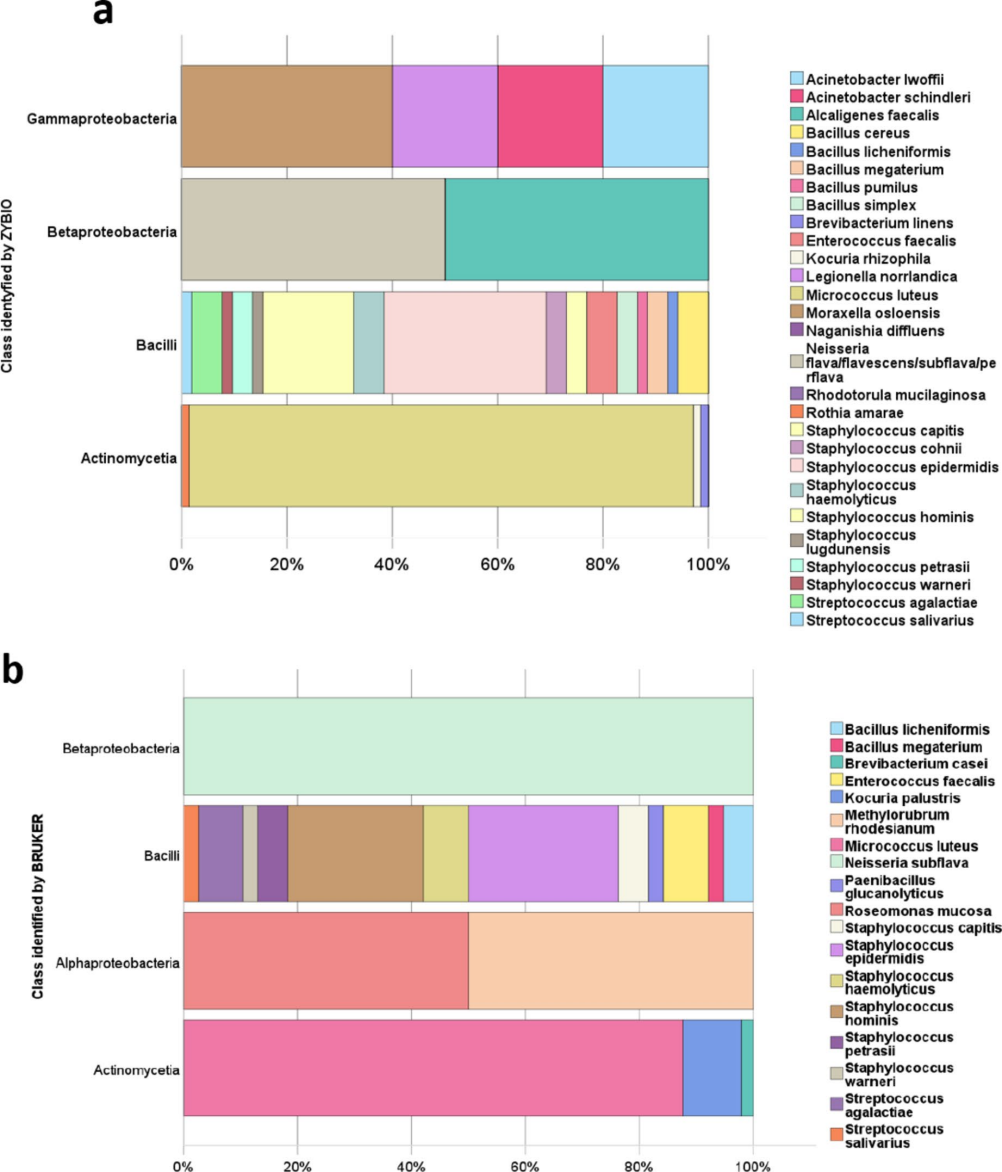
Comparison of species identified by MALDI systems categorized by classes of identified microorganisms. (a) species identified by the Zybio system, (b) species identified by the Bruker system.

Among the 48% species identifications by the Zybio system, the most numerous and heterogeneous classes were Actinomycetia and Bacilli, respectively. In the Bacilli class, 16 different species were identified, with *Staphylococcus epidermidis* (*N* = 16) and *Staphylococcus hominis* (*N* = 9) being the most represented. The Actinomycetia class was the most homogeneous, predominantly represented by *Micrococcus luteus* (*N* = 67) (Fig. 4a). Among the 33% species identifications by the Bruker system, the *Bacilli* class was the most heterogenic with 12 species (Fig. 4b). The most prevalent species in this class were *Staphylococcus epidermidis* (*N* = 10) and *Staphylococcus hominis* (*N* = 9). 88% of the identified microorganisms from the Actinomycetia class was recognized as *Micrococcus luteus* (*N* = 43).

## Correlation analysis between the bacteria species and the physicochemical parameters of fuels

**Fig. 5:**
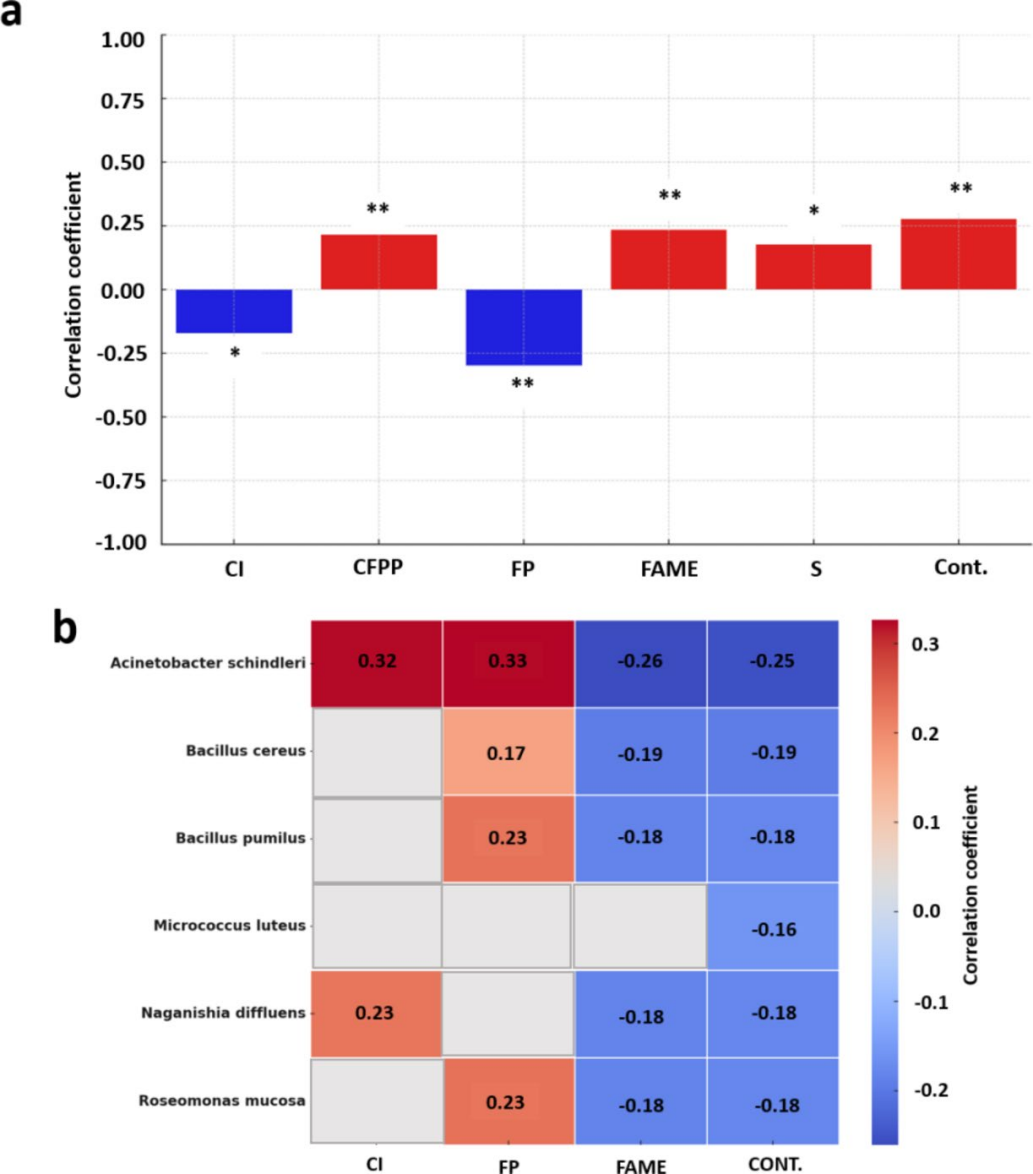
Correlation analysis of the presence (a) and occurrence of specific bacterial species (b) with the physicochemical parameters of fuels. (a) Pearson correlation coefficient values for the presence of bacteria with various physicochemical parameters, with positive correlations shown by red bars and negative correlations by blue bars. The significance level was set at 0.05 and is indicated on the graph: as ** (p-values < 0.01), * (p-values < 0.05). (b) Statistically significant Pearson correlation coefficient values for specific bacterial species with physicochemical parameters. The colour gradient correlates with the value of the correlation coefficient. (CI) Cetane Index, (FP) Flash Point, (CFPP) Cold Filter Plugging Point, FAME (Fatty Acid Methyl Esters), (Cont) Contaminants, (S) sulfur content.

Correlation analysis revealed significant relationships between the presence of bacteria and the physicochemical parameters of fuels (Fig. 5a). The strongest statistically significant positive correlations were found between the bacteria present and contaminant content (0.2765, *p* = 0.0005), fatty acid methyl esters (FAME) content (0.2355, *p* = 0.0031), and cold filter plugging point (CFPP) (0.2146, *p* = 0.0071). The strongest statistically significant negative correlation was observed for the flash point (FP) (-0.2971, *p* = 0.0002). Additionally, specific bacterial species were found to significantly correlate with selected physicochemical parameters (Fig. 5b). Negative correlations were demonstrated for *Acinetobacter schindleri*,* Bacillus cereus*,* B. pumilus*,* Naganishia diffluens*, and *Roseomonas mucosa* with FAME content and contaminants in the fuel. A strong positive correlation was observed for *Acinetobacter schindleri* with the Cetane Index (CI) and FP, as well as for *B. pumilus* and *Roseomonas mucosa* with FP.

### Chemical profiles of the diesel fuel

**Fig. 6:**
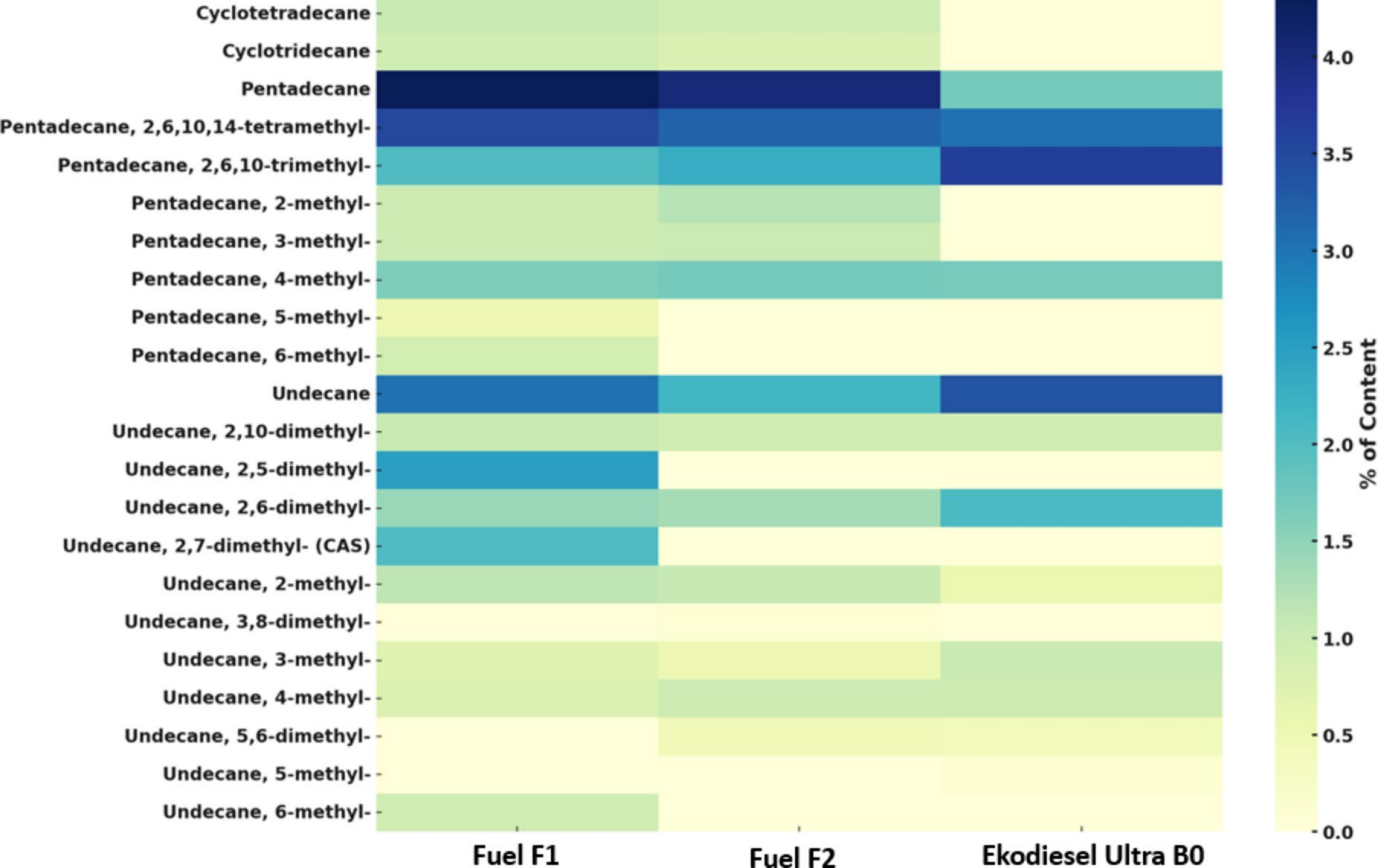
The heatmap of the selected compounds identified as unique in the fuel samples compared to the standard fuel sample. The relative percentage content of each compound is correlated with the color intensity.

Differences in the chemical profiles of the three diesel fuel types compared to a standard reference sample were demonstrated (Fig. 6). Notably, 25 unique compounds specific to each fuel type were identified in Ekodiesel Ultra B0, 42 in F1, and 45 in F2. Among the identified compounds were isomers of pentadecane, undecane, hexa-tetradecane, cyclotridecane, and cyclotetradecan, whose occurrence and the average percentage content varied across the different fuel types. The distinct differences in the percentage content of pentadecane and its isomers were observed with specific isomers such 5-methyl, and 6-methyl detected exclusively only in fuel F1.

## The identification mismatches in two MALDI systems

The observed discrepancies in score values and reference databases between the two MALDI systems affect the identification mismatches (Supp. Figure 6, Supp. Figure 5). Among high-confidence identifications, both Zybio and Bruker systems unequivocally identified 24% of the species submitted to identification, including *Staphylococcus hominis*,* Staphylococcus epidermidis*,* and Streptococcus agalactiae* (Supp. Figure 6a). For high-confidence identifications by Bruker, the Zybio system assigned lower score values ranging from 1.37 to 1.82, which prevented species-level identification by the Zybio system and generated mismatch identification by Zybio system (Supp. Figure 5, Supp. Figure 6b). Most mismatch identification were noted for the species *Micrococcus luteus*. The Bruker system identified *M. luteus* in seven samples, whereas the Zybio system was able to determine only the genus in four cases, failed to identify species in two cases, and identified the genus Legionella in one case (Supp. Figure 6b). Among the five high-confidence identifications of *Kocuria palustris* by Bruker, the Zybio system identified only the genus Kocuria sp. in two cases, and failed to identify species or genus in the remaining cases. For other species, such as *Methylorubrum rhodesianum* and *Neisseria subflava*, the Zybio system did not identify any species (Supp. Figure 6b). Among low-confidence identifications by Bruker, the Zybio system assigned higher score values for 33% and lower score values for 16% of the samples compared with Zybio. 21% of the samples were identified with similar score values by both MALDI systems (Supp. Figure 6b).

## The comparison of MALDI and 16 S rRNA sequencing in the identification of microorganisms from fuels

To verify and confirm the identification of microorganisms, 16 S rRNA sequencing was performed (Table 1, Supp. Tab. S2). The 16 S rRNA analysis confirmed the proteomic identification for certain species unequivocally identified by both MALDI systems, such as *Staphylococcus hominis* (99.51% identity) and *Micrococcus luteus* (99.49% identity). When the MALDI analysis could distinctly indicate the genus, e.g., *Bacillus sp.*, *Staphylococcus sp.*, *Pseudarthrobacter sp.*, the 16 S rRNA analysis enabled species-level identification, respectively, as *Bacillus cereus* group (99.93% identity), *Staphylococcus hominis* (99.57% identity), and *Pseudarthrobacter equi* (99.57% identity). Sequencing-based analysis facilitated the confirmation of inconsistent identifications between the two MALDI systems, validating Bruker’s identification for *Micrococcus luteus*, where the Zybio system failed to identify any species. Some genetic and proteomic identifications were discordant, indicating genus and species misidentification by the MALDI system. For example, *Micrococcus luteus* was identified by the Bruker system, while 16 S analysis indicated *Peribacillus sp.* (99.93/99.79% identity); similarly, *Bacillus megaterium* was identified as *Priestia sp.* (100/99.86% identity), and *Naganishia sp.* was identified as *Methylorubrum aminovorans* (99.93% identity).

**Table 1:** The result of bacteria identification based on two MALDI systems (Bruker, Zybio) and 16 S rRNA sequencing. The identification levels are marked by colors as follows: green- species identification (score ≥ 2.000), yellow- genus identification (score 1.700–1.999), red- no identification (score 0.000–1.699). The results of 16 S rRNA sequencing were presented after comparing the sequences with the most related sequences available in the NCBI database, along with the indicated level of identity.

BRUKER	ZYBIO	16s rRNA	The most related species from NCBI	IDENTITY %
*Staphylococcus hominis*	*Staphylococcus hominis*	*Staphylococcus hominis*	*Staphylococcus hominis* subsp. *novobiosepticus* GTC 1228	99.51
			*Staphylococcus hominis* DM 122	99.51
*Micrococcus luteus*	*Micrococcus luteus*	*Micrococcus luteus*	*Micrococcus yunnanensis* YIM 65,004	99.71
			*Micrococcus luteus* NCTC 2665	99.49
			*Micrococcus luteus* DSM 20,030	99.49
*Bacillus sp.*	*Bacillus sp.*	*Bacillus cereus* group sp.	*Bacillus cereus* ATCC 14,579	99.93
			*Bacillus cereus* IAM 12,605	99.93
			*Bacillus cereus* JCM 2152	99.93
*Staphylococcus* sp.	*Staphylococcus* sp.	*Staphylococcus hominis*	*Staphylococcus hominis* subsp. *novobiosepticus* GTC 1228	99.57
			*Staphylococcus hominis* DM 122	99.50
P*seudarthrobacter* sp.	*Pseudarthrobacte*r sp.	*Pseudarthrobacter equi*	*Pseudarthrobacter equi IMMIB L-*1606	99.57
*Micrococcus luteus*	no identification	*Micrococcus luteus*	*Micrococcus yunnanensis* YIM 65,004	99.71
			*Micrococcus luteus* NCTC 2665	99.43
*Micrococcus luteus*	no identification	*Micrococcus luteus*	*Micrococcus yunnanensis* YIM 65,004	99.78
*Micrococcus luteus*	no identification	*Peribacillus* sp.	*Peribacillus frigoritolerans* DSM 8801	99.93
			*Peribacillus simplex* LMG 11,160	99.79
			*Peribacillus simplex* NBRC 15,720 = DSM 1321	99.79
*Bacillus megaterium*	*Bacillus megaterium*	*Priestia* sp.	*Priestia aryabhattai* B8W22	100
			*Peribacillus acanthi* L28	99.93
			*Priestia megaterium* NBRC 15,308 = ATCC 14,581	99.86
			*Priestia megaterium* ATCC 14,581	99.79
*Naganishia* sp.	*Naganishia diffluens*	*Methylorubrum aminovorans*	*Methylorubrum aminovorans* JCM 8240	99.93
			*Methylorubrum extorquens* TK 0001	99.70
			*Methylorubrum suomiense* NCIMB 13,778	99.56

## Discussion

In our study, we identified the microbiological contaminants in three types of diesel fuel storage tanks using two MALDI-TOF MS systems (Bruker Daltonik GmbH and Zybio Inc) for the detection and characterization of these microorganisms, with the identification validated by 16 S rRNA gene sequencing. The study demonstrated that the quantity of identified microorganisms varies depending on the type of fuel in the storage tank (Fig. 1a). The highest number of microorganisms was observed in the tank with Ekodiesel Ultra B0 fuel. Ekodiesel Ultra B0 is a fuel devoid of biocomponents. Fuel F1 contains additives that enhance operational properties, including lubricants and detergents. Similar to Fuel F2, it contains up to 7% biocomponents (FAME). Floyd et al., 2022 demonstrated that the microbiological composition in fuel tanks varies depending on the fuel components, indicating a positive correlation between the quantity of *Trichomaceae* and the content of FAME^8^. Additionally, pure biodiesel showed higher microbial contamination compared to enriched fuel blends^7^. This is likely due to the absence of water-binding biocomponents in Ekodiesel Ultra B0. In their absence, water can accumulate in the tank, promoting microbial growth. Additionally, it is devoid of antioxidant additives, leading to conditions that favour the development of microorganisms. FAME can exhibit biocidal properties, reducing the microbial population, which supports our observation that the lowest number of microorganisms was found in diesel fuel. Among the three types of analyzed fuels, the highest number of microorganisms was identified in the bottom part of the Fuel F1 tank (Fig. 1a). Similarly, the greatest contamination was observed by Rodríguez-Rodríguez et al., 2009 at the bottom of crude oil tanks, especially when water was accumulated in the tank^2^. Therefore, despite the high-quality enhancing additives in Fuel F1, water from condensation, leaks, or contaminants during refuelling was accumulated at the bottom of the tank, promoting the proliferation of microorganisms. In addition, mechanical impurities, such as dust particles in contact with the surfaces of the installation, and organic residues accumulating at the bottom of the tank, provide an excellent nutrient source for microorganisms, supporting their growth. The most abundant sources of microbial isolates collected from fuel tanks were the middle parts of Fuel F1 and Fuel F2 (Fig. 1b), likely due to the optimal conditions at the water-fuel phase boundary, which is rich in both nutrients and water.

Among all the identified microorganisms in the fuels, the majority belong to environmental bacteria (Fig. 4), which was also noticed by Gaylarde et al., 1999; Komariah et al., 2022 ^1,7^. Aerobic or facultatively anaerobic bacteria dominated among the microorganisms found in diesel fuel primarily due to their metabolic flexibility and adaptability to variable conditions commonly found in fuel storage systems^2^. Oxygen is present in the surface layers of fuel tanks, supporting the growth of bacteria. The metabolic versatility of facultatively anaerobic bacteria allows them to survive and proliferate in fluctuating oxygen conditions typically found in fuel tanks^2,18^. In addition, aerobic and facultatively anaerobic bacteria often demonstrate robust survival mechanisms that allow them to endure the harsh chemical environment of diesel fuel, which is toxic to many microorganisms. In fuel tanks, microaerophilic conditions occurring, especially at the fuel-water interface, promote the growth of facultatively anaerobic bacteria^18,19^. Our study indicated the predominance of Gram-positive bacteria in diesel fuel classified into two the most represented classes of *Actinomycetia* and *Bacilli* (Supp. Figure 3b, Fig. 4a) similar to the observation of^2,5,6,20^. Ganesh & Lin, 2009 found that Gram-positive bacteria were effective in diesel degradation and biosurfactant production^20^. Vidal-Verdú et al., 2022 identified Gram-positive bacteria from the genera *Staphylococcus* and *Bacillus* predominating in the microbiota below the lids of diesel fuel deposits. Additionally, Suflita et al., 2012 reported that Gram-positive bacteria, particularly from the Firmicutes group, including *Bacillus*, *Lactobacillus*, and *Streptococcus*, were predominant in diesel fuel^6^. Bacteria from the classes *Actinomycetia* and *Bacilli* are known for their ability to biodegrade petroleum hydrocarbons and survive harsh environmental conditions through the production of endospores. These microorganisms can originate from various stages of fuel production, transport, and storage^6^. For the species *Bacillus cereus* isolated from oil-contaminated soils, the ability to degrade petroleum has been demonstrated^15^. The biodegradative properties of a wide range of refinery products (diesel oil, crude oil, kerosene, and used engine oil) have been shown for *Bacillus cereus* Strain DRDU1 by Borah & Yadav, 2014^21^. Species from the genus *Bacillus* have also been described as capable of synthesizing lipopeptide biosurfactants that degrade hydrocarbons by up to 88% ^22^. The species *Brevibacterium* from the class *Actinomycetia* has been investigated for its ability to emulsify and utilize diesel fuel, demonstrating efficient utilization of this oil^23^. Therefore, the microorganisms isolated in our studies offer promising prospects for future research focused on the bioremediation of contaminated environments and the discovery of new strains capable of biofuel production.

Our study revealed significant relationships between the presence of bacteria and the physicochemical parameters of fuels. The strongest positive correlations were found between bacterial presence and contaminant content, FAME content, and cold filter plugging point (CFPP) (Fig. 5). Floyd et al., 2022 demonstrated that members of the fungal family *Trichomaceae* dominate in fuels with higher FAME content^8^. The high hygroscopicity and biodegradability of FAME make fuels enriched with this biocomponent more susceptible to microbial contamination^24^. Increased microbial proliferation in diesel supplemented with FAME was observed by Bobić Vedranka et al., 2015^4^. Moreover, Lai et al., 2014 found a significant linear correlation between increasing CFPP values and higher contents of saturated fatty acid methyl esters in biodiesel^3^. Therefore, microbial contamination can affect fuel properties related to flow and filtration, which are critical for maintaining proper CFPP parameters^25^.

Aliphatic and cycloaliphatic hydrocarbons were detected with varied percentage content in fuel samples (Fig. 6). The identified hydrocarbons are characteristic of diesel fuels and play critical roles in improving cetane number, stabilization of the fuel mixture and reduction of the mechanical wear acting as lubricity additives. Among the identified compounds were isomers of pentadecane, undecane, hexa-tetradecane, cyclotridecane, and cyclotetradecane. Notably, some compounds, such as branched alkanes (e.g., pentadecane, 5-methyl-) and cycloalkanes (e.g., cyclotridecane, cyclotetradecane), could potentially be associated with microbial activity. As highlighted by Heider et al.^26^, biodegradation processes performed by hydrocarbon-degrading bacteria (e.g., *Rhodococcus*,* Alcanivorax*,* Pseudomonas*), particularly under oxygen-limited conditions, can lead to the production of identified compounds via the breakdown of larger hydrocarbon molecules. Similarly, Wilkes et al.^27^ describe the widespread ability of bacteria to degrade alkanes, including noticed in our study undecane and hexadecane. Cycloaliphatic compounds such as cyclotridecane, while generally more resistant to biodegradation, can also be metabolized by bacteria specialized in cycloaliphatic hydrocarbon degradation through both aerobic and anaerobic pathways^28^. The differences in relative abundance and the percentage composition of specific hydrocarbons in analyzed diesel fuel can serve as indirect evidence of varying levels of microbial activity or differences in the microbial communities. However, to conclusively determine whether the identified compounds in the fuel samples originate from bacterial metabolism or are intrinsic to the diesel fuel, further analyses—such as isotopic labeling or metabolic profiling—are required.

Our study attempted to apply the MALDI-TOF MS technique for rapid and sensitive identification of microorganisms in fuel and tank sediments based on the analysis of unique microbial protein patterns. This method has found broad applications in clinical diagnostics, epidemiological studies, antibiotic resistance detection, and pathogen identification in various clinical and environmental matrices^29–31^. Our study demonstrates that the MALDI technique can be a valuable tool for detecting microbial contamination in fuels. The use of two MALDI systems (Bruker MALDI Biotyper^®^ and Zybio MALDI-TOF MS) significantly enhanced the benefits in the context of detecting microbial contamination in fuels, especially since both systems showed identification differences at the genus and species levels (Fig. 2a) what was also noticed by^16^. Additionally, statistically significant differences in the score values for the most numerous classes of microorganisms (*Actinomycetia* and *Bacilli*) between the two identification systems were demonstrated in our studies (Fig. 3a). The Zybio system identified 48% of all microorganisms at the species level, whereas the Bruker system identified 33%. When considering genus-level identification only, the Bruker system was more effective (24% compared to 16% with the Zybio system). However, when all isolates identified to the species level (which inherently include genus-level identification) are accounted for, the overall genus-level identification was more effective with the Zybio system, achieving 64% compared to 57% with the Bruker system (Fig. 2a). Result of our study indicate that the reference mass spectra abundance of the library and the representation of species in the library plays a pivotal role in the MALDI identification of the microorganisms since the commercial platforms for automatic microbial IDs (MBT Biotyper and EXS2600) used “library-based approach” for microorganisms taxonomy^32^. Especially since the classification of the microorganisms relies on the match score of the obtained MS profile of the isolate to the MS profile in the reference library. However, the reference spectral databases used by MALDI-TOF MS platforms are primarily optimized for clinical isolates, resulting in a greater number of reference spectra for these strains. In our study, isolated strains were environmental microorganisms. Studies comparing the effectiveness of the MALDI method in identifying clinical and environmental bacteria clearly indicate a significant effect of the bacterial living environment on the reliability of obtained identification results. Research on bacteria such as the *Bacteroidetes fragilis* group, *Burkholderia cepacia* complex, or *Virgibacillus* sp., comparing the influence of isolate source (clinical vs. environmental), demonstrates that the accuracy of taxonomic assignment for bacterial isolates derived from human material consistently and significantly surpassed that of isolates from other environmental sources^33^. This dependence is associated with the fact that bacteria occurring in different environments may demonstrate diverse, distinct phenotypic characteristics. Consequently, their identification based on protein profiles using the MALDI-TOF MS technique may be hindered due to the presence in the obtained MS spectra of not only signals from ribosomal proteins but also those appearing in connection with adaptive mechanisms of bacterial response to stress conditions. Additionally, environmental samples exhibit significantly higher species biodiversity compared to clinical samples, which adds complexity to the identification process^34^. However, the discrepancies in identifications observed between the two MALDI-TOF MS systems can be attributed not only to the absence of specific MS spectra in the reference database of one system but also to differences in the origins of the bacterial isolates used to construct their respective reference databases. Notably, the EXS 2600 system relies on a reference database developed using its proprietary collection of microbial isolates, predominantly sourced from China. The methodology used to prepare and input spectra of reference strains is also important for the consistency and accuracy of the identification. The MBT Biotyper and EXS 2600 systems employ distinct criteria for signal inclusion and validation^35^. In Biotyper system signal must be present in at least 25% of the acquired spectra during the identification process to be deemed significant and included in the reference database. The Biotyper system incorporates approximately 70 significant signals per reference spectrum, resulting in a broader and more diverse database. In the Zybio platform, a signal must be detected in at least 50% of the acquired spectra to be considered significant for inclusion in the reference database. This higher threshold ensures greater signal consistency but results in a leaner database, with fewer signals per reference spectrum compared to the Biotyper system. However, by expanding the reference libraries to include specific microorganisms associated with fuels, utilizing industrial research data, and applying advanced bioinformatics technologies, both systems could better meet the specific requirements of microbiological diagnostics in the fuel industry^2^. This is particularly important as our studies have shown that the type of fuel from which isolates were obtained affects the sensitivity of identification (Fig. 2b).

The observed discrepancies in score values and reference databases between the two MALDI systems, Zybio and Bruker, significantly contribute to identification mismatches. Only 24% of the species were unequivocally identified by both systems (Suppl. Figure 5, Supp. Figure 6). Misidentifications of mycobacteria by MALDI-TOF MS have been reported by Rodriguez-Temporal et al., 2022 even at high-level identification^17^. These incorrect identifications result from nearly identical protein profiles of closely related species^17^. For example, Rodriguez-Temporal et al. analyzed clinical bacterial isolates using a Microflex spectrometer (Bruker Daltonics) and the Vitek 2 system, achieving a 73% concordance in identifications between the two systems. One of the primary challenges in using MALDI-TOF MS for environmental bacteria is the lack of comprehensive reference databases that include spectra from a wide variety of environmental strains. This limitation can result in misidentifications or low-confidence matches for uncommon or novel species^36^. The specificity and accuracy of identification can be enhanced by implementing custom libraries tailored to specific environments or microbial communities. Studies by Gupta et al., 2023 have demonstrated that incorporating local isolates into the reference database significantly improves the system’s ability to accurately identify environmental bacteria^37^. To enhance the confidence of identification, 16 S rRNA sequencing was performed (Table 1, Supp. Table 2). The 16 S rRNA sequencing confirmed the proteomic identifications by the MALDI systems for certain species, such as *Micrococcus luteus* and *Staphylococcus hominis*, with high accuracy. For samples where MALDI analysis could only resolve the genus level, such as *Bacillus* sp., *Staphylococcus* sp., and *Pseudarthrobacter* sp., the 16 S rRNA sequencing provided more precise species-level identifications. For example, *Bacillus* sp. was identified as part of the *Bacillus cereus* group, and *Staphylococcus* sp. was confirmed as *Staphylococcus hominis*. Woo et al., 2003 reported that the MicroSeq 500 16 S rRNA-based bacterial identification system was effective in identifying clinically significant bacterial isolates with ambiguous biochemical profiles, achieving 81.1% success in species-level identification which is often a limitation for MALDI-TOF MS^38^. Some genetic identifications did not match the proteomic identifications, indicating potential issues in genus and species identification by the MALDI system, as observed in our study for *Micrococcus luteus*, *Bacillus megaterium*, and *Naganishia sp* (Fig. 5a; Table 1). Similar misidentifications were demonstrated for *Lactobacillus* isolates by Anderson et al., 2014, highlighting the need for expanding reference databases^39^. Additionally, 16 S rRNA sequencing did not fully resolve problems related to intra- and interspecies classification, as noted by Angolini et al.^11^. The results of our study clearly indicate that the observed misidentifications were not always attributable to the absence of reference spectra in the libraries of the investigated systems. For instance, an isolate identified using 16 S rRNA sequencing as *Paenarthrobacter nitroguajacolicus* was not identified by both MALDI systems even though spectra of *P. nitroguajacolicus* were present in Biotyper and EXS2600 libraries. A similar situation applies to *Curtobacterium flaccumfaciens*, *Peribacillus* sp., *Micrococcus* sp. However, we observed instances where misidentifications could be attributed to the absence of corresponding reference spectra in the databases, as exemplified by *Priestia flexa*,* Pseudarthrobacter equi*, and *Methylorubrum aminovorans*. Nonetheless, such cases represented a minority of the overall misidentifications. The investigation of the two different bacterial identification approaches enhanced the accuracy of species-level identifications in fuels and resolved inconsistencies, thereby ensuring a higher level of confidence in the MALDI analysis.

## Conclusion

Our study indicates that MALDI-TOF MS can be effectively employed to monitor changes in microbial communities within fuel storage tanks over time, providing a proactive tool for identifying emerging contamination issues before they lead to significant fuel degradation or system damage. While MALDI-TOF MS offers substantial benefits for rapid bacterial identification, identifying environmental bacteria remains challenging due to the diversity and complexity of microbial communities in fuels. Continuous enrichment and updating of reference databases, sample preparation protocols, and methodological adjustments are essential to enhance the accuracy and reliability of this technology in environmental microbiology. Leveraging the capabilities of MALDI technology can significantly improve the detection, identification, and management of microbiological contamination in fuels, thereby enhancing fuel quality, system reliability, and operational efficiency.

## Materials and methods

### Material source

The fuel samples were collected on September 6, 2022, and October 7, 2022, from four different storage tanks containing various types of diesel fuel located at fuel stations (Supp. Figure 1). The tanks varied in the type of fuel they stored and included Ekodiesel Ultra B0, Fuel1 (F1) and Fuel2 (F2). Fuel samples numbered 1–4 (250 mL) were taken from the upper and lower parts of 24- and 28-year-old tanks (DPPL1 and DPPL2, respectively), which contained Ekodiesel Ultra B0. This fuel is basic diesel fuel with additives used during formulation, including cetane enhancers, biocides, and a multifunctional package containing detergents, antioxidants, antifoaming agents, lubricity additives, and conductivity improvers. The level and type of tank from which the sample 1–4 was taken was as follows: 1: DPPL1 (lower part of the tank), 2: DPPL1 (upper part of the tank), 3: DPPL2 (lower part of the tank), 4: DPPL2 (upper part of the tank). The tank was located at fuel station 1362 Konin, Poland. Samples numbered 5–7 (250 mL) were taken from different parts of a Fuel F2 tank storing. The level and type of tank from which the sample 5–7 was taken was as follows: 5: Fuel F2_2 (lower part of the tank), 6: Fuel F2_3 (lower part of the tank), 7: Fuel F2_4 (lower part of the tank). Samples numbered 8–16 (250 mL) were taken from a 28-year-old tank and an internal sampler containing Fuel F1 and Fuel F2. The level and type of tank from which the sample 8–16 was taken was as follows: 8: Fuel F1_3 (lower part of the tank), 9: Fuel F1_1 (lower part of the tank), 10: Fuel F1_2 (lower part of the tank), 11: Fuel F1 (sample taken before tank cleaning), 12: Fuel F2_1 (lower part of the tank), 13: Fuel F1_4 (lower part of the tank), 14: Fuel F1_sampler, 15: Fuel F2_sampler, 16: Fuel F2 (sample taken before tank cleaning). The tank was located at fuel station 1495 Bydgoszcz, Poland.

### Physicochemical analyses of fuel samples

Physicochemical analyses of the fuel samples were conducted by Orlen Laboratory (Płock, Poland) (Supplementary Table S1). The Cetane Index (CI) was determined according to PN-EN ISO 4264:2018-08, and the Cold Filter Plugging Point (CFPP) was measured in accordance with PN-EN 116:2015-09. The Flash Point (FP), a critical parameter in the characterization and safety assessment of diesel fuel, was determined according to PN-EN ISO 3015:2019-06. The percentage content of Fatty Acid Methyl Esters (FAME) was measured in compliance with PN-EN 14078:2014-06. The Sulfur Content (S) was assessed according to PN-EN ISO 20846:2020-03, and the Contaminant Content was evaluated following PN-EN 12662:2014-05.

### Gas chromatography

Gas chromatography-time-of-flight mass spectrometry (GC-TOF/MS) analysis was conducted using a 6890 N gas chromatograph (Agilent Technologies, Waldbronn, Germany) coupled with a TOF mass spectrometer (Leco, Michigan, USA). The system was equipped with an Equity-1 capillary column (30 m × 0.25 mm × 0.25 μm, Supelco, Germany). The oven temperature program was as follows: an initial temperature of 40 °C, held for 3 min, was ramped at 20 °C/min to 300 °C, and maintained for 10 min. The injector was operated in split-splitless mode at a temperature of 280 °C. Before the analysis, the samples were filtered using a PF filter, diluted at a 1:100 ratio in hexane, and 1 µL of the prepared solution was injected into the chromatographic system. The analysis results were compared with a standard fuel sample (Merck, Germany). Mass spectrometric analyses were performed in full-scan mode across a mass-to-charge ratio (m/z) range of 35–550. Spectra were acquired using electron ionization (EI) at 70 eV. The ion source temperature was maintained at 225 °C, while the transfer line temperature was set to 280 °C. Chromatographic data acquisition and processing were performed using ChromaTOF software (Leco).

### Bacteria cultures

**Fig. 7:**
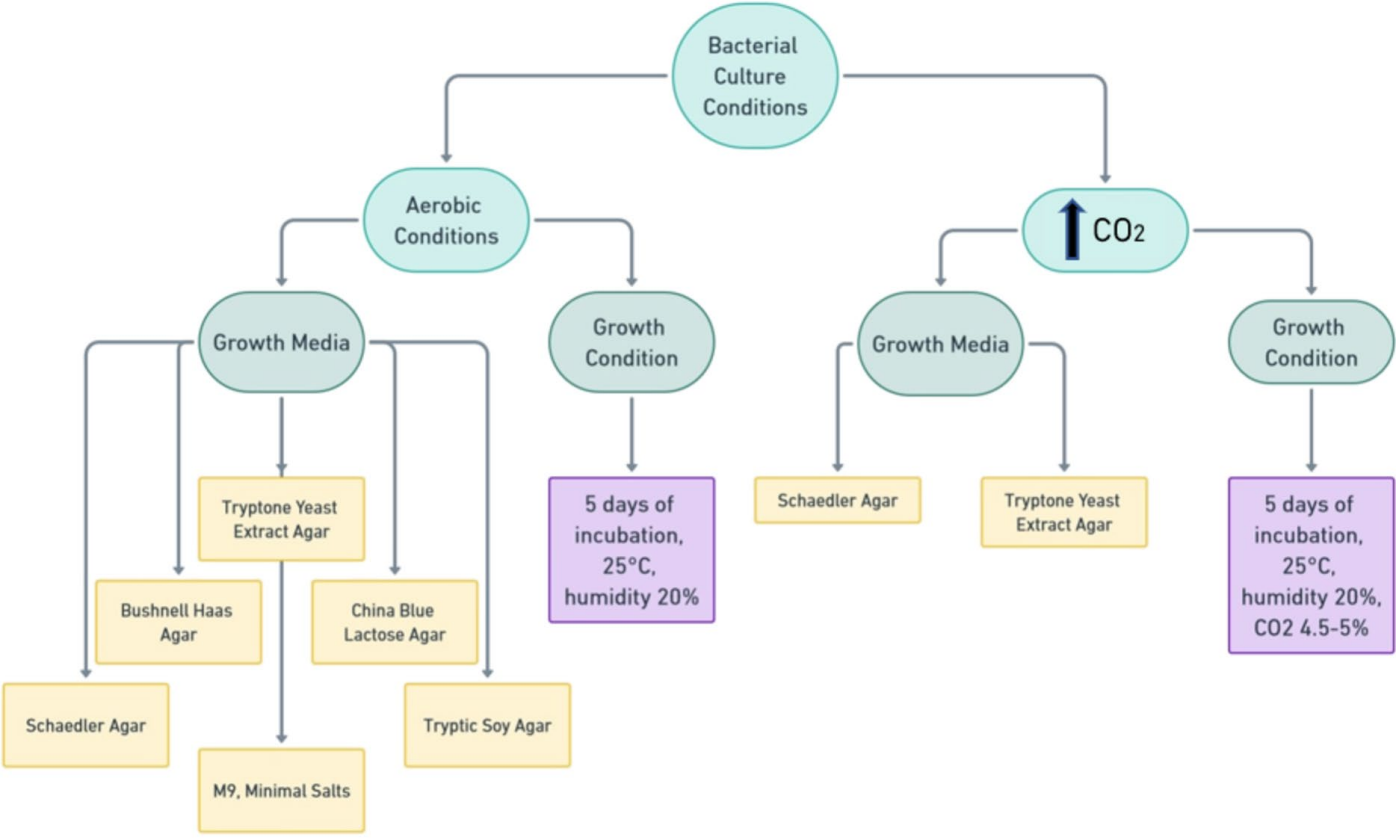
Schematic representation of the bacterial culture conditions.

From each fuel, samples were taken from the top, middle, and bottom parts of the collection bottle (Supp. Figure 2). Then, collected samples were plated in triplicate by mixing 100 µl of fuel samples with 10 ml of liquid medium before solidification. The bacteria were cultured under aerobic conditions and elevated CO_2_ concentrations (5% CO2) in six and two different media, respectively. The media and culture conditions are presented in Fig. 7. After 5 days of incubation, the streak plate method was used to obtain pure cultures. For this purpose, single colonies were transferred to TYEA medium enriched with sterile diesel fuel (1%). After 24 h of culturing, the bacteria were identified using MALDI-TOF MS.

### Preparation of bacterial extracts

From selected distinct bacterial colonies, cell biomass was collected using a sterile microbial loop, and protein extraction using a formic acid/acetonitrile mixture in a ratio of 1:1 was performed. The details of the extraction procedure were described by Sibińska et al., 2024). Then, 1 µL of the protein extracts was spotted onto a MALDI plate (MTP 384 target plate polished steel BC, Bruker Daltonik GmbH, Germany), dried at room temperature, and then overlaid with 1 µL of the matrix α-CHCA (α-cyano-4-hydroxycinnamic acid) dissolved in a standard solvent solution consisting of HPLC-grade water, trifluoroacetic acid, and acetonitrile in percentages of 47.5%, 2.5%, and 50%, respectively, to a final concentration of 10 mg/ml.

### MALDI-TOF MS analysis

The prepared samples were analyzed using a Microflex LT MALDI–TOF/TOF mass spectrometer (Bruker Daltonik GmbH, Germany) and an EXS2600 MALDI-TOF MS (Zybio Inc., Chongqing, China) with a linear TOF mass analyzer and positive ionization mode. The obtained mass spectra of bacterial protein profiles underwent smoothing, baseline correction, and calibration against the BTS standard using flexAnalysis software (MALDI Biotyper 4.1 platform, Bruker Daltonik GmbH) and System software Ex-Accuspec version V1 for Bruker and Zybio, respectively^40^.

Mass spectra were analyzed in a mass-to-charge ratio (m/z) range of 2000 to 20,000 for both MALDI systems. The identification of bacterial isolates was achieved by comparing the obtained mass spectra with reference spectra of microorganisms in the databases of both MALDI platforms: the Bruker Biotyper 4.1 software and library (version rev. H/2021), with 10,834 entries (Bruker Daltonics), and Mass Spectrometry System Software Ex-Accuspec version V1 (Zybio)^35^.

The identification scores were interpreted according to the manufacturer’s recommendations: a score of 2.000 or above signified species-level identification, a score between 1.700 and 1.999 suggested identification at the genus level, and a score below 1.700 was considered a failure in identification.

### Identification of selected bacterial strains by PCR

The identification of the selected bacterial strains was based on the sequencing of the 16 S rRNA gene. The isolation of genomic DNA was conducted using the E.Z.N.A.^®^ Bacterial DNA Kit (Omega Bio-tek, Norcross, US), adhering to the supplied protocol with minor modifications. Specifically, the incubation of the bacterial suspension, after the addition of lysozyme, was carried out for 30 min (37 °C), followed by a subsequent 30-minute incubation with lysostaphin (37 °C).

The assessment of the quantity and quality of the isolated DNA was performed spectrophotometrically using a UV‒Vis NanoDrop 2000c spectrophotometer (Thermo Fisher Scientific). Samples exhibiting an A_260/280_ ratio within the range of 1.8 to 2.0 were selected for PCR amplification. The amplification of the 16S rRNA utilized the primers 27F (5’-AGA GTT TGA TCC TGG CTC AG-3’) and 1492r (5’-GGT TAC CTT GTT ACG ACT T-3’), as outlined by Gardes and Bruns (1993) and White et al. (1990). The PCR reactions were facilitated by the Taq PCR Master Mix (2×) (Qiagen), with each reaction containing 10 ng of DNA. The cycling conditions were as follows: an initial denaturation at 94 °C for 3 min; followed by 30 cycles of denaturation at 94 °C for 30 s, annealing at 55 °C for 30 s, and extension at 72 °C for 90 s; with a final extension at 72 °C for 7 min.

The analysis of the presence and size of the PCR products was conducted on a 1.0% w/v agarose gel, which was stained with SYBR™ Green I Nucleic Acid Gel Stain (Invitrogen). Sequencing of the 16 S rRNA fragments utilized the 27 F and 1492r universal primers^41^. The assembly of the forward and reverse reads was performed using BioEdit software (version 7.2.5). The consensus sequences were compared against the GenBank rRNA database for identification. A threshold of at least 99% coverage and 99% similarity was applied for accurate taxonomic assignment. All sequenced DNA fragments were submitted to the GenBank database (see Data availability statement).

### Statistical analysis

Data analysis and chart generation were executed utilizing the advanced capabilities of the PS IMAGO PRO 9.0 package (version 29.0.0.0., Predictive Solutions, Poland), an extensive suite integrated with IBM SPSS Statistics. Additionally, Python (version 3.8) was employed in conjunction with the Pandas library (version 1.2.0) and Matplotlib (version 3.4.0).

Chi-square tests and pairwise chi-square tests with Bonferroni correction were conducted to determine whether the observed differences in the number of cultured bacteria on various media were statistically significant and to identify how the groups varied from each other. The Wilcoxon signed-rank test was performed to assess whether the number of identifications at the species level, genus level and the number of non-identifications differed significantly between the two MALDI systems. A standard significance threshold (α = 0.05) was adopted for all analyses.

To evaluate differences in “score” values for each bacterial class between the two MALDI systems, the non-parametric Mann-Whitney U test was employed due to the varying sample sizes within different bacterial classes. Correlation analysis of bacterial types with physicochemical fuel parameters was performed using Pearson’s correlation coefficient, utilizing the Pandas library. Pearson’s correlation significance tests identified which correlations were statistically significant at the 0.05 significance level.

## Electronic supplementary material

Below is the link to the electronic supplementary material.

Supplementary Material 1

## Data Availability

Sequence data that support the findings of this study have been deposited in the GenBank database, with the public URL for the sequence collection https://www.ncbi.nlm.nih.gov/sites/myncbi/1F1i8raBplj5d/collections/64187410/public/. GenBank accession numbers: PP833142.1, PP837814.1, PP837806.1, PP859244.1, PP859243.1, PP859242.1, PP859241.1 , PP859240.1 PP859239.1, PP837823.1, PP837822.1, PP837821.1, PP837820.1, PP837819.1, PP837818.1, PP837817.1PP837816.1, PP837815.1, PP859490.1, PP859486.1PP859487.1, PP859485.1, PP859484.1 PP859483.1, PP859263.1, PP859262.1, PP859261.1, PP859260.1, PP859258.1, PP859254.1, PP859253.1, PP859252.1PP859251.1, PP859250.1 PP859249.1, PP859248.1, PP859247.1, PP859246.1.
