# Supplementary material for: Comparative analysis of microbial contamination in diesel fuels using MALDI-TOF MS
Source: Sci Rep. 2025 Feb 6;15:4525. doi: 10.1038/s41598-025-87713-1 (PMC11802864; doi:10.1038/s41598-025-87713-1)
Supplement: Supplementary file 1 — Supplementary Material 1 [file 41598_2025_87713_MOESM1_ESM.pdf]

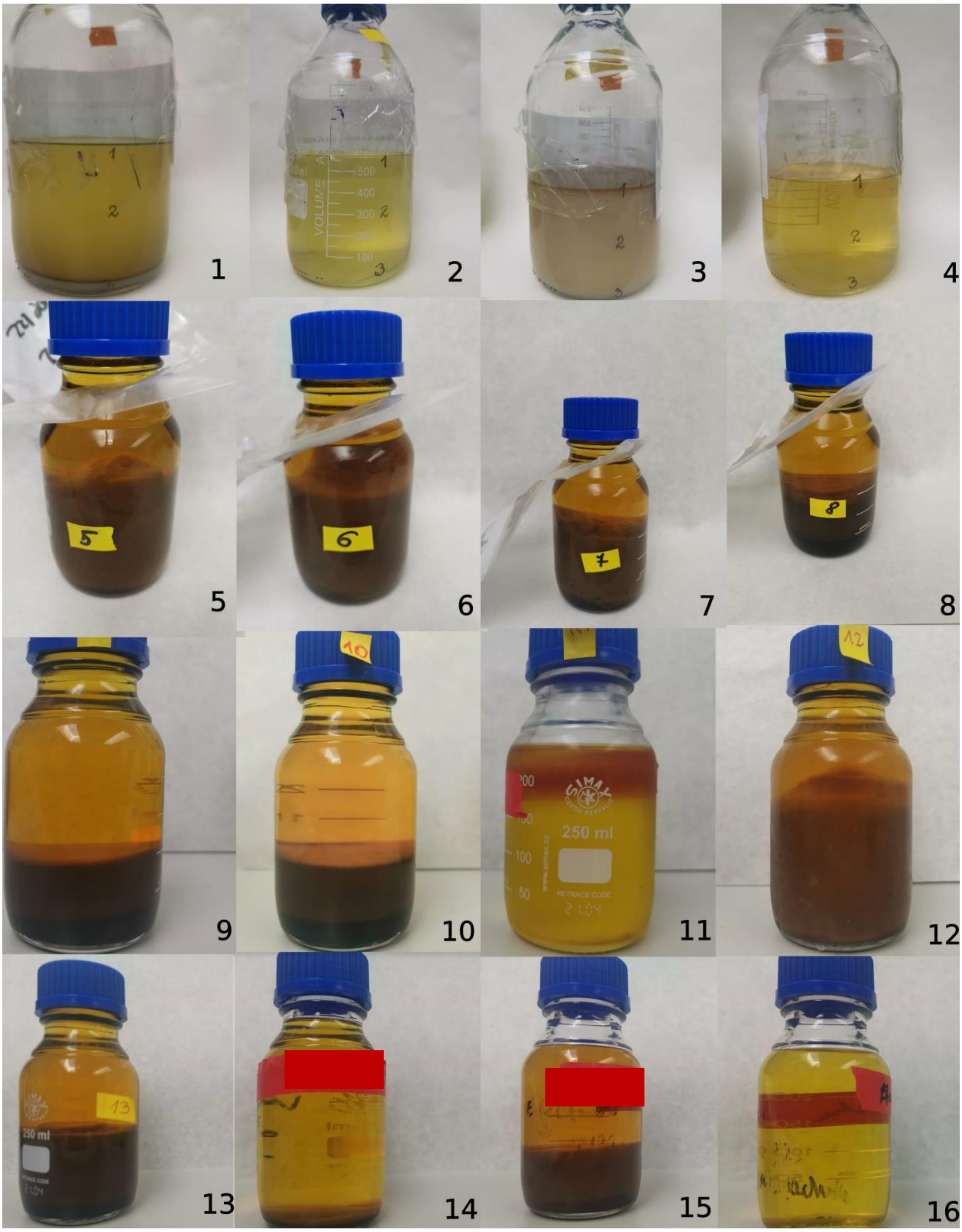

Supplementary Figure S1. Appearance of fuel samples after collection from respective tank levels. The numbers correspond to samples taken from various parts of the storage tanks with three types of fuels: 1: DPPL1 (lower part of the tank), 2: DPPL1 (upper part of the tank), 3: DPPL2 (lower part of the tank), 4: DPPL2 (upper part of the tank), 5: Fuel F2_2 (lower part of the tank), 6: Fuel F2_3 (lower part of the tank), 7: Fuel F2_4 (lower part of the tank), 8: Fuel F1_3 (lower part of the tank), 9: Fuel F1_1 (lower part of the tank), 10: Fuel F1_2 (lower part of the tank), 11: Fuel F1 (sample taken before tank cleaning), 12: Fuel F2_1 (lower part of the tank), 13: Fuel F1_4 (lower part of the tank), 14: Fuel F1_sampler, 15: Fuel F2_sampler, 16: Fuel F2 (sample taken before tank cleaning).

Supplementary Table S1. The results of the physicochemical analyses of the fuel samples.

Parameters \ Type of fuel	Ekodiesel ultra B0	Fuel F1	Fuel F2
**CI -** Cetane Index	54.6	53.1	53.7
	55.5	53.4	52.7
	54.7	53.2	53.7
**CFPP -** Cold Filter Plugging Point °C	-11	-9	-3
	-10	-9	-4
	-11	-9	-3
**FAME -** Fatty Acid Methyl Esters %(V/V)	0	6.8	6.9
	0	6.8	6.9
	0	6.8	6.9
**Sulfur Content** mg/kg	8	7.3	8.6
	6.6	7.2	7.7
	6.9	7.0	8.3
**Contaminant Content** mg/kg	6	12	13.5
	6	10	12
	7.8	12	12.5

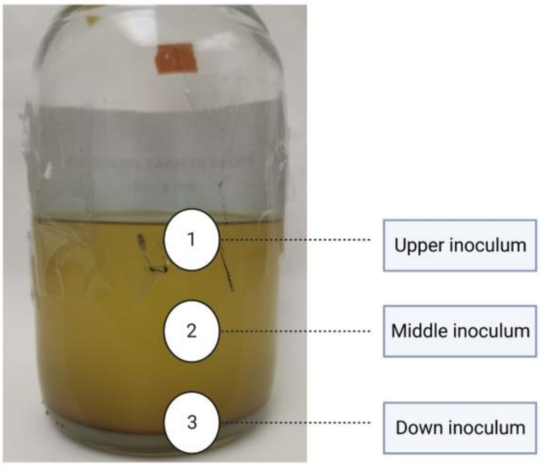

Supplementary Figure S2. Method of bacterial inoculum collection for bacterial cultures. The three different batches of bottles from which inoculated were taken were marked.

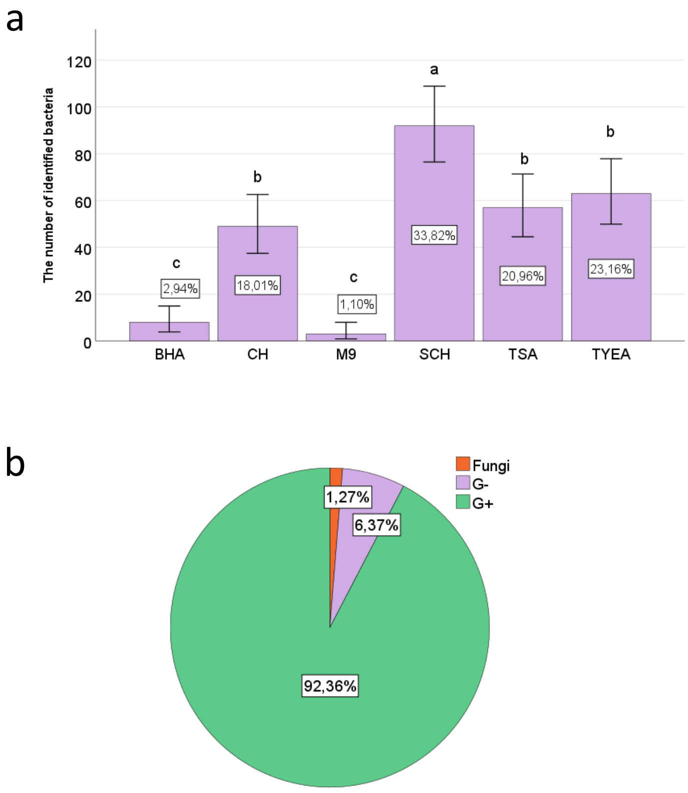

Supplementary Figure S3. Growth of microorganisms in various culture media (a) and the types of identified microorganisms categorized into gram-positive bacteria (G+), gram-negative bacteria (G-), and fungi (b). Results of the post-hoc pairwise chi-square test with Bonferroni correction are presented with different letters for the groups differed statistically significantly at a significance level of 0.05.

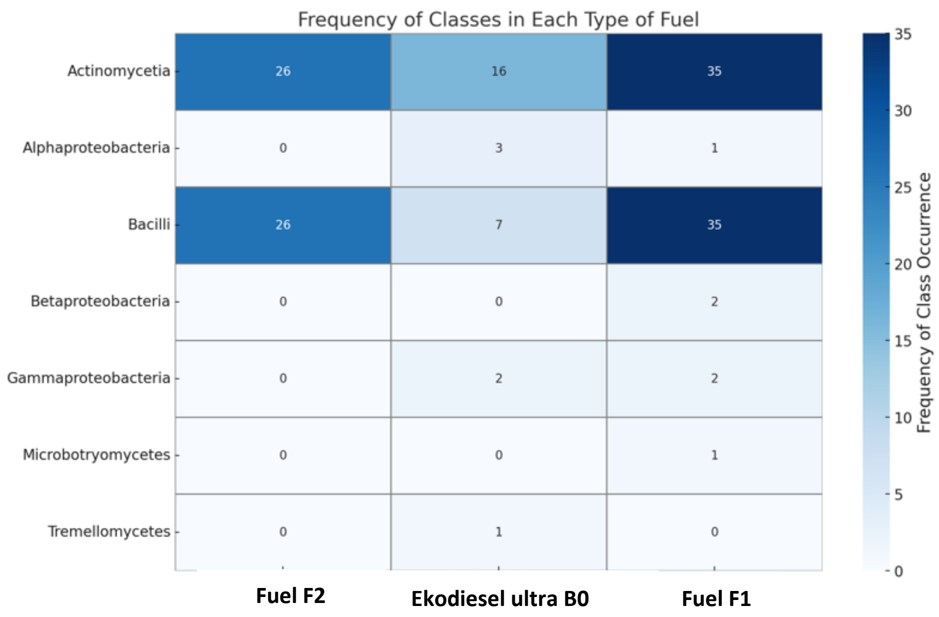

Supplementary Figure S4. Heatmap of the frequency of occurrence of various classes of microorganisms in different fuel types. Colour intensity correlates with the number of microorganisms within each class.

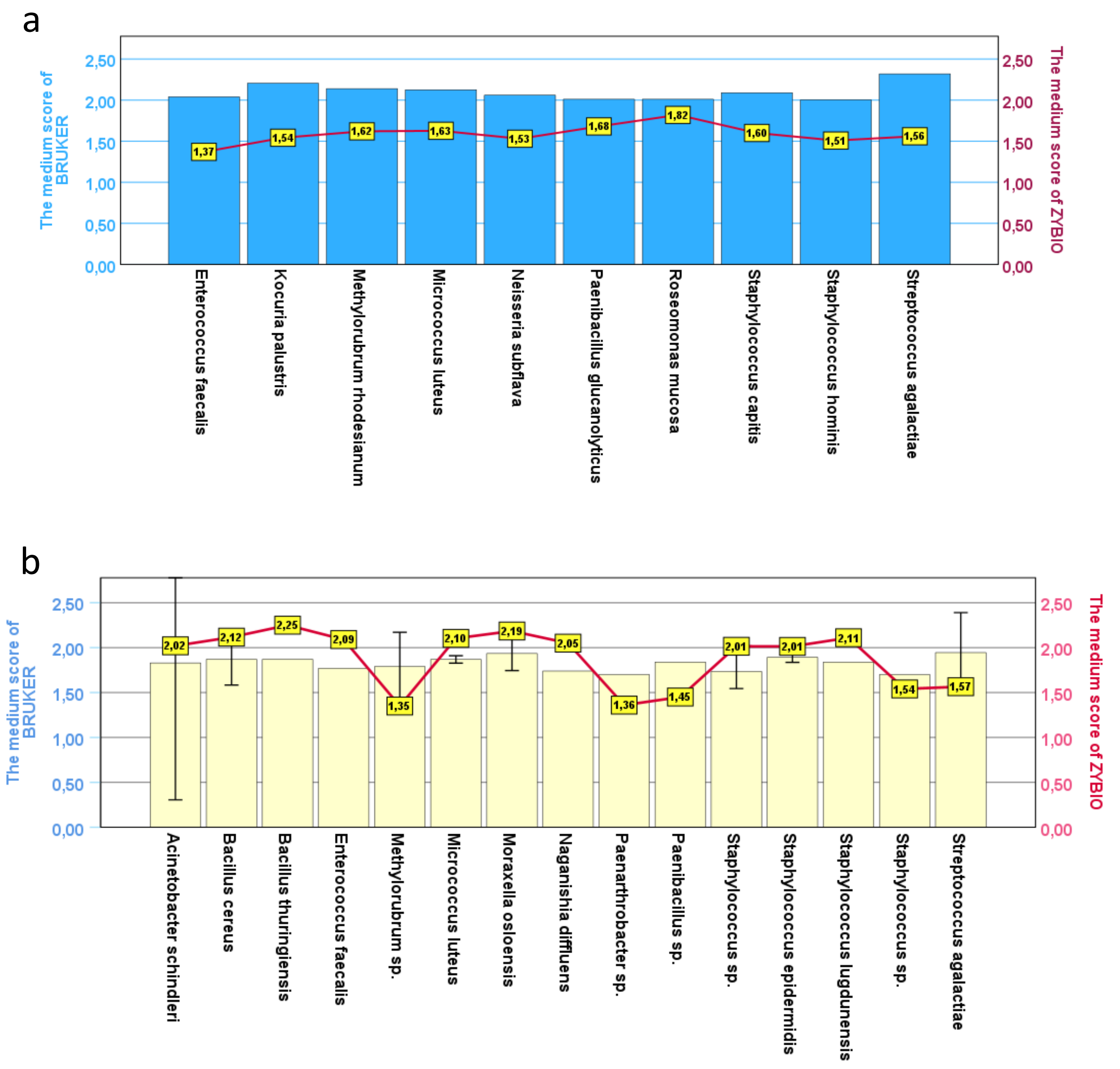

Supplementary Figure S5. The differences in the score values between two MALDI systems identified at high (a) and low (b) levels of identification assigned to Bruker identification. (a) Comparison of score value differences for high-confidence identifications by the Bruker and Zybio systems, (b) Comparison of score value differences for low-confidence identifications by the Bruker and Zybio systems. The bar charts represent score values for the Bruker system. Score values for the Zybio system are provided in yellow boxes with a trend line.

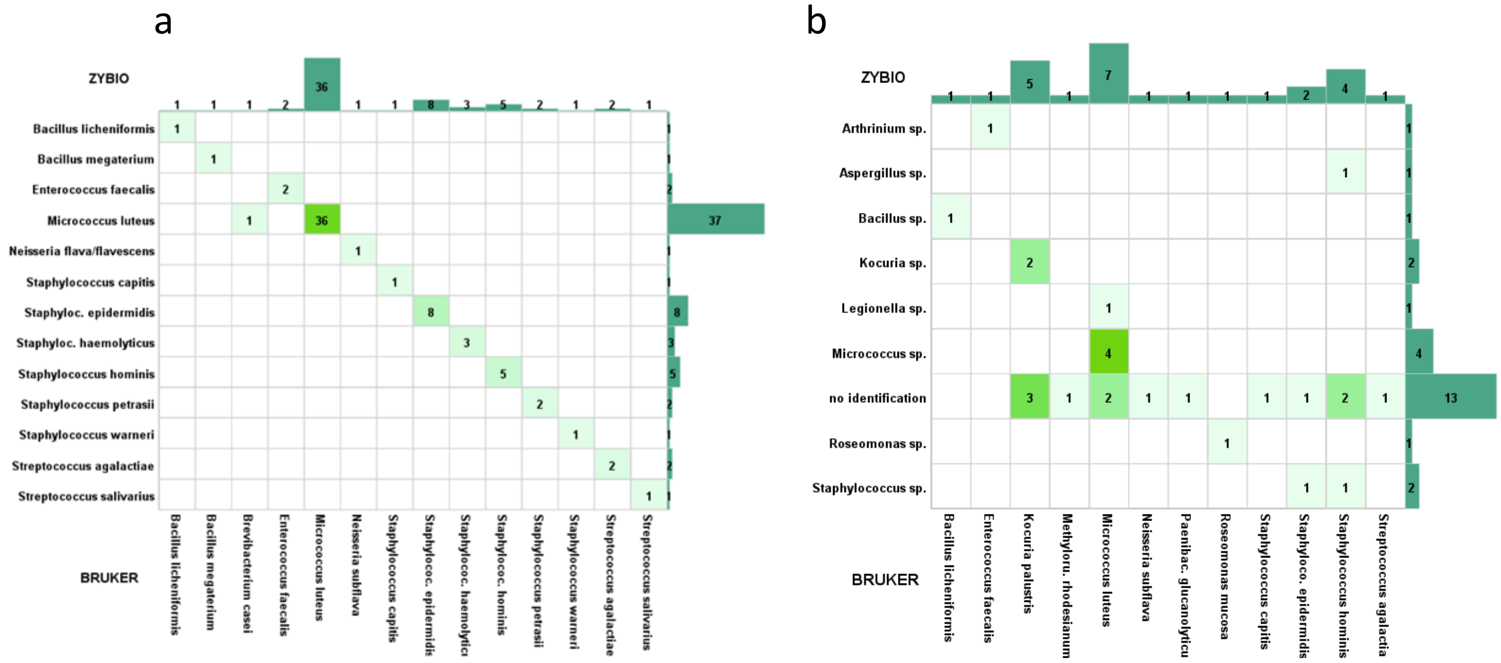

Supplementary Figure S6. Identification matches at the species level (a) and mismatches identified in Bruker high-identification vs. Zybio (b). The colours correlate with the number of bacteria identified by Zybio (the left part of the heat map) and Bruker (the bottom part of the heat map).

Supplementary Table S2. The result of bacteria identification based on two MALDI systems (Bruker, Zybio) and 16S rDNA sequencing. The identification levels of MALDI are marked by colors as follows: green- species identification (score ≥ 2.000), yellow- genus identification (score 1.700–1.999), red- no identification (score 0.000–1.699). The results of 16S rDNA sequencing were presented after comparing the sequences with the most related sequences available in the NCBI database, along with the indicated level of identity.

BRUKER	ZYBIO	16s rDNA	The most related species from NCBI	IDENTITY %
Micrococcus luteus	*Micrococcus sp.*	*Micrococcus luteus*	Micrococcus yunnanensis YIM 65004	99.86
			Micrococcus aloeverae AE-6	99.85
			Micrococcus luteus NCTC 2665	99.64
Bacillus sp.	*Bacillus sp.*	*Bacillus cereus* group sp.	Bacillus cereus ATCC 14579	99.93
			Bacillus cereus IAM 12605	99.93
			Bacillus cereus JCM 2152	99.93
Micrococcus luteus	*Micrococcus luteus*	*Micrococcus luteus*	Micrococcus yunnanensis YIM 65004	99.78
			Micrococcus luteus NCTC 2665	99.56

			Micrococcus luteus DSM 20030	99.56
Micrococcus luteus	no identification	*Peribacillus sp.*	Peribacillus frigoritolerans DSM 8801	99.93
			Peribacillus simplex LMG 11160	99.79
			Peribacillus simplex NBRC 15720 = DSM 1321	99.79
Staphylococcus capitis	*Lactobacillus sp.*	*Staphylococcus epidermidis*	Staphylococcus epidermidis Fussel	99.93
			Staphylococcus epidermidis NBRC 100911	99.86
Micrococcus luteus	*Micrococcus luteus*	*Micrococcus luteus*	Micrococcus luteus strain NCTC 2665 Micrococcus terreus strain V3M1	99.50 97.91
Bacillus sp.	*Bacillus* sp.	*Bacillus cereus* group sp.	Bacillus thuringiensis IAM 12077	99.57
			Bacillus thuringiensis ATCC 10792	99.57
			Bacillus thuringiensis NBRC 101235	99.50
			Bacillus paranthracis MCCC 1A00395	99.50
Micrococcus luteus	no identification	*Micrococcus luteus*	Micrococcus yunnanensis YIM 65004	99.71
			Micrococcus luteus NCTC 2665	99.43
Bacillus megaterium	*Bacillus megaterium*	*Priestia sp.*	Priestia aryabhattai B8W22	100
			Peribacillus acanthi L28	99.93
			Priestia megaterium NBRC 15308 = ATCC 14581	99.86
			Priestia megaterium ATCC 14581	99.79
Micrococcus luteus	*Micrococcus luteus*	*Micrococcus luteus*	Micrococcus yunnanensis YIM 65004	99.71
			Micrococcus luteus NCTC 2665	99.49
			Micrococcus luteus DSM 20030	99.49
Micrococcus luteus	no identification	*Micrococcus luteus*	Micrococcus yunnanensis YIM 65004	99.78

Micrococcus luteus	*Micrococcus* sp.	*Micrococcus luteus*	Micrococcus yunnanensis YIM 65004	99.56
			Micrococcus luteus NCTC 2665	99.35
			Micrococcus luteus DSM 20030	99.35
Bacillus sp.	*Bacillus sp.*	*Bacillus cereus* group sp.	Bacillus cereus ATCC 14579	100
			Bacillus cereus IAM 12605	100
			Bacillus cereus JCM 2152	100
			Bacillus cereus CCM 2010	100
			Bacillus cereus NBRC 15305	100
			Bacillus tropicus MCCC 1A01406	99.93
			Bacillus proteolyticus MCCC 1A00365	99.93
			Bacillus nitratireducens MCCC 1A00732	99.93
Peribacillus sp.	*Bacillus* sp.	*Peribacillus* sp.	Peribacillus frigoritolerans DSM 8801	99.93
			Peribacillus simplex LMG 11160	99.72
			Peribacillus simplex NBRC 15720 = DSM 1321	99.72
			Peribacillus muralis LMG 20238	99.57
Staphylococcus hominis	*Staphylococcus hominis*	*Staphylococcus hominis*	Staphylococcus hominis subsp. novobiosepticus GTC 1228	99.51
			Staphylococcus hominis DM 122	99.51
no identification	*Bacillus* sp.	*Peribacillus* sp.	Peribacillus frigoritolerans DSM 8801	99.93
			Peribacillus simplex LMG 11160	99.79
			Peribacillus simplex NBRC 15720 = DSM 1321	99.79
			Peribacillus muralis LMG 20238	99.58
			Peribacillus frigoritolerans DSM 8801	99.86

no identification	*Bacillus* sp.	*Peribacillus* sp.	Peribacillus simplex LMG 11160	99.65
			Peribacillus simplex NBRC 15720 = DSM 1321	99.44
			Peribacillus muralis LMG 20238	99.44
Staphylococcus sp.	*Staphylococcus* sp.	*Staphylococcus hominis*	Staphylococcus hominis subsp. novobiosepticus GTC 1228	99.57
			Staphylococcus hominis DM 122	99.50
Micrococcus luteus	*Micrococcus luteus*	*Micrococcus luteus*	Micrococcus yunnanensis YIM 65004	99.71
			Micrococcus luteus NCTC 2665	99.49
			Micrococcus luteus DSM 20030	99.49
Bacillus licheniformis	*Bacillus* sp.	*Bacillus subtilis* group sp.	Bacillus licheniformis ATCC 14580	99.93
			Bacillus licheniformis DSM 13	99.93
			Bacillus licheniformis BCRC 11702	99.93
			Bacillus licheniformis NBRC 12200	99.72
			Bacillus haynesii NRRL B-41327	99.72
Methylorubrum sp.	no identification	*Priestia flexa*	Priestia flexa NBRC 15715	100
			Priestia flexa IFO15715	100
Bacillus sp.	no identification	*Bacillus pumilus*	Bacillus pumilus strain NBRC 12092	99.57
			Bacillus zhangzhouensis MCCC 1A08372	99.50
Paenibacillus glucanolyticus	no identification	*Bacillus pumilus*	Bacillus pumilus NBRC 12092	100
			Bacillus zhangzhouensis MCCC 1A08372	99.93
			Bacillus stratosphericus 41KF2a	99.65
Rhodotorula sp.	*Rhodotorula sp.*	*Pseudarthrobacter equi*	Pseudarthrobacter equi IMMIB L-1606	99.00

Paenibacillus glucanolyticus	no identification	*Bacillus pumilus*	Bacillus pumilus NBRC 12092	100
			Bacillus zhangzhouensis MCCC 1A08372	99.93
Methylorubrum sp.	no identification	*Micrococcus endophyticus*	Micrococcus endophyticus YIM 56238	98.54
Pseudarthrobacter sp.	*Pseudarthrobacter* sp.	*Pseudarthrobacter equi*	Pseudarthrobacter equi IMMIB L-1606	99.57
Naganishia sp.	*Naganishia diffluens*	*Methylorubrum aminovorans*	Methylorubrum aminovorans JCM 8240	99.93
			Methylorubrum extorquens TK 0001	99.70
			Methylorubrum suomiense NCIMB 13778	99.56
no identification	no identification	*Paenarthrobacter nitroguajacolicus*	Paenarthrobacter nitroguajacolicus strain G2-1	100
			Paenarthrobacter aurescens strain DSM 20116	99.64
no identification	no identification	*Pseudarthrobacter equi*	Pseudarthrobacter equi strain IMMIB L-1606	99.35
Methylorubrum sp.	no identification	*Priestia flexa*	Priestia flexa NBRC 15715	99.93
			Priestia flexa IFO15715	99.93
no identification	no identification	*Peribacillus sp.*	Peribacillus simplex strain LMG 11160	100
			Peribacillus simplex NBRC 15720 = DSM 1321	100
			Peribacillus frigoritolerans DSM 8801	99.72
			Peribacillus frigoritolerans DSM 8801	99.72
			Peribacillus muralis LMG 20238	99.51
Methylorubrum rhodesianum	no identification	*Priestia flexa*	Priestia flexa NBRC 15715	100
			Priestia flexa IFO15715	100
Staphylococcus hominis	*Staphylococcus hominis*	*Bacillus pumilus*	Bacillus pumilus NBRC 12092	99.93
			Bacillus zhangzhouensis MCCC 1A08372	99.86

			Bacillus stratosphericus 41KF2a	99.58
Staphylococcus sp.	*Staphylococcus hominis*	*Staphylococcus hominis*	Staphylococcus hominis subsp. novobiosepticus GTC 1228	99.79
Staphylococcus sp.	*Staphylococcus hominis*	*Paenibacillus glucanolyticus*	Paenibacillus glucanolyticus NBRC 15330	99.79
			Paenibacillus glucanolyticus DSM 5162	99.72
Staphylococcus sp.	*Staphylococcus hominis*	*Micrococcus luteus*	Micrococcus yunnanensis YIM 65004	99.49
Naganishia sp.	*Naganishia diffluens*	*Curtobacterium flaccumfaciens*	Curtobacterium flaccumfaciens BCCM/LMG 3645	100
			Curtobacterium oceanosedimentum ATCC 31317	99.50
